# Opening the Sacred Chamber: The Cultural and Ethical Odyssey of Cardiac Surgery

**DOI:** 10.3390/jcdd12100378

**Published:** 2025-09-24

**Authors:** Vasileios Leivaditis, Georgios Mavroudes, Francesk Mulita, Nikolaos G. Baikoussis, Athanasios Papatriantafyllou, Vasiliki Garantzioti, Konstantinos Tasios, Levan Tchabashvili, Dimitrios Litsas, Paraskevi Katsakiori, Stelios F. Assimakopoulos, Konstantinos Nikolakopoulos, Andreas Antzoulas, Elias Liolis, Spyros Papadoulas, Efstratios Koletsis, Manfred Dahm

**Affiliations:** 1 Department of Cardiothoracic and Vascular Surgery, Westpfalz Klinikum, 67655 Kaiserslautern, Germany; vnleivaditis@gmail.com (V.L.); thanospap9@yahoo.gr (A.P.); mdahm@westpfalz-klinikum.de (M.D.); 2 School of Social Anthropology, University of Sussex, Falmer, Brighton BN1 9QU, UK; mavroudis79@hotmail.com; 3 Department of General Surgery, General Hospital of Eastern Achaia—Unit of Aigio, 25100 Aigio, Greece; tchabashvili.alexander@gmail.com; 4 Department of Cardiac Surgery, Ippokrateio General Hospital of Athens, 11527 Athens, Greece; nikolaos.baikoussis@gmail.com; 5 Department of Surgery, General University Hospital of Patras, 26504 Patras, Greece; vigarant@yahoo.com (V.G.); kostastasiosmd@gmail.com (K.T.); dimlitsas@icloud.com (D.L.); vkatsak@gmail.com (P.K.); a.antzoulas@hotmail.com (A.A.); 6 Division of Infectious Diseases, Department of Internal Medicine, Medical School, University of Patras, 26504 Patras, Greece; sassim@upatras.gr; 7 Department of Vascular Surgery, General University Hospital of Patras, 26504 Patras, Greece; konstantinosn@yahoo.com (K.N.); spyros.papadoulas@gmail.com (S.P.); 8 Department of Oncology, General University Hospital of Patras, 26504 Patras, Greece; lioliselias@yahoo.gr; 9 Department of Cardiothoracic Surgery, General University Hospital of Patras, 26504 Patras, Greece; ekoletsis@hotmail.com

**Keywords:** cardiac surgery, medical ethics, symbolism of the heart, history of medicine, bioethics, cultural anthropology, transplantation ethics

## Abstract

Cardiac surgery, now a routine medical intervention, was once deemed unthinkable—not merely due to technical limitations, but because of deep-seated cultural, religious, and philosophical prohibitions. This article traces the historical and ethical trajectory of the human heart from a sacred, inviolable symbol of the soul to a surgically accessible organ. Through an interdisciplinary lens that integrates medical history, anthropology, theology, and contemporary bioethics, we examine how shifts in metaphysical belief, technological progress, and moral reasoning gradually legitimized cardiac intervention. From ancient Egyptian funerary rites and classical cardiocentric models to medieval religious taboos and Enlightenment redefinitions of the body, the heart’s transformation reflects broader changes in how humanity conceives life, death, and identity. The emergence of modern cardiac surgery, especially heart transplantation and extracorporeal technologies, raised new ethical dilemmas, challenging the boundaries between tissue and meaning, biology and personhood. This study argues that despite its clinical secularization, the heart retains a unique symbolic gravity that continues to shape public perception and professional responsibility. In the age of precision medicine, cardiac surgery remains not only a technical act but an existential gesture—a transgression that demands both scientific mastery and moral reverence.

## 1. Introduction

Across centuries and civilizations, the heart has remained both a corporeal and transcendent emblem—a site where anatomy and myth converge. Unlike other organs, which could be dissected or removed with relative detachment, the heart was revered as sacred—its role suspended between sustaining life and signifying it. While other organs, such as the liver in both Egyptian and Greco-Roman traditions, also bore profound cultural and spiritual meanings [1], the heart uniquely combined these metaphysical associations with an unparalleled centrality in moral, emotional, and religious frameworks. This dual positioning of the heart, as both biological and symbolic, has been extensively examined in the fields of medical humanities and cardiocentric anthropology [2].

Perennially regarded as the core of one’s being, the heart was imbued with moral, spiritual, and cosmological value. This is evident in diverse cultural traditions—from the ancient Egyptian ritual of weighing the heart in the Book of the Dead, to the Christian iconography of the Sacred Heart, where it symbolizes divine love and suffering [3,4,5,6]. The historical resistance to cardiac intervention cannot be fully explained by technical limitations alone; rather, it was shaped by profound ontological taboos that rendered the heart inviolable.

For much of human history, the heart was not simply a vital organ—it was the epicenter of emotion, intellect, and soul. In ancient Egypt, it was believed to house the soul and serve as the basis for moral judgment in the afterlife. In Greco-Roman thought, the heart was likewise conceived as the seat of thought and emotion. These enduring perceptions reinforced the heart’s central role in shaping human identity and spirituality. As Figueredo argues, such historical beliefs continue to influence contemporary symbolic representations of the heart, even as modern medicine has recast it as a mechanical pump [7,8].

This article traces the long arc of the heart’s cultural and medical transformation—from its sacred untouchability to its surgical accessibility. By exploring the intertwined genealogies of religious doctrine, philosophical imagination, and medical ethics, this inquiry aims to reveal the historical process by which cardiac intervention became morally and practically permissible. While cultural and religious prohibitions undoubtedly played a pivotal role in rendering the heart untouchable for much of history, they were not the sole determinants of surgical inaccessibility. Technical limitations, including the absence of anesthesia, antiseptic techniques, and reliable hemostasis, also made cardiac intervention nearly inconceivable, even in societies or periods where metaphysical prohibitions were less pronounced. Accordingly, this article does not argue that symbolic or cultural barriers were universally exclusive causes, but rather that they formed a dominant and enduring layer of resistance which, when combined with technical constraints, delayed the advent of cardiac surgery. Throughout this trajectory, it becomes however evident that the heart’s “sacredness” functioned as an epistemological stance—one that shaped perception and inhibited action, even when the imperative to save a life was clear. The act of cutting into the heart thus emerges as not merely a technical breakthrough, but a transgression historically encoded with existential and ethical weight.

The present review focuses primarily on Western cultural, religious, and scientific traditions in order to trace the dominant historical and ethical trajectories that shaped the perception of the heart and delayed the advent of cardiac surgery. We acknowledge that rich perspectives on the heart also existed within Eastern medical and philosophical systems, such as those of China, India, and Japan. However, these traditions fall beyond the scope of this article, which is centered on developments within Western medicine.

## 2. Materials and Methods

This study adopts a qualitative, interdisciplinary methodology, combining historical analysis, philosophical reflection, and bioethical inquiry. The research is situated within the framework of medical humanities and draws upon diverse fields, including history of medicine, religious studies, classical philosophy, anthropology, and cardiology.

A hermeneutic approach was employed to interpret primary texts within their respective historical, cultural, and symbolic contexts. Additionally, a genealogical method, inspired by the work of Michel Foucault, was used to trace the shifting meanings and ethical implications of cardiac intervention across time—from antiquity to the modern era.

### Source Selection and Search Strategy

Relevant literature was identified through structured and manual searches in academic databases such as PubMed, JSTOR, Google Scholar, and historical archives. Bibliographic tracing of cited works in key publications was also used to broaden the source base.

Inclusion Criteria:

- Peer-reviewed journal articles, academic books, and authoritative historical texts.
- Primary sources from antiquity to the modern period (e.g., Aristotle, Galen, Vesalius, Harvey, sacred scriptures).
- Scholarly interpretations from reputable disciplines such as medical history, ethics, theology, and cultural anthropology.
- Contemporary studies exploring the symbolic, emotional, or ethical dimensions of the heart or cardiac surgery.

Exclusion Criteria:

- Non-scholarly or non-peer-reviewed publications, such as popular media articles or unverified web sources.
- Texts lacking relevance to the symbolic, ethical, or historical framing of the heart or cardiac surgery.
- Clinical case reports or technical studies unrelated to the broader conceptual or cultural analysis.

This approach aims not to provide empirical generalizations but rather to illuminate the shifting moral, metaphysical, and epistemological landscapes that have governed the human relationship with the heart—particularly as they pertain to the rise in cardiac surgery as both medical practice and existential gesture.

## 3. Ancient and Classical Views: The Heart as the Sacred Center of Being

### 3.1. The Heart in Greek Philosophy and Medicine

In the foundational texts of classical philosophy, the heart was conceptualized primarily as a sovereign locus of animation, intellect, and will, with its biological functions relegated to the margins. Aristotle, writing in the 4th century BCE, proposed in On the Parts of Animals that the kardia (heart) was the origin of sensation and the instigator of movement, whereas the brain merely served as a cooling mechanism. Although this theory was eventually displaced by anatomical discoveries, it cemented the heart’s centrality in Western metaphysical thought—not as a mechanical organ, but as the primal seat of life. This cardiocentric primacy was more than symbolic; it structured the entire diagnostic imagination of early Western medicine [9,10].

The Hippocratic Corpus, compiled roughly between the 5th and 4th centuries BCE, also remained within the bounds of this cardiocentric model. While it introduced systematic observations on the pulse and correlations with humoral imbalances, it did not deviate far enough to consider the heart a surgically accessible organ. The very notion of opening the heart remained culturally unthinkable—a medical impossibility not due to technical inability alone, but because of deeply embedded taboos. Recent scholarship confirms that resistance to cardiac intervention was driven more by cosmological and metaphysical fears than by a lack of anatomical knowledge or surgical skill [2,11].

Later, in the 2nd century CE, Galen of Pergamon introduced a more physiologically oriented vision of the heart’s function. In his seminal work On the Usefulness of the Parts of the Body, he described the heart as the furnace of innate heat and the distributor of pneuma, the life-giving spirit carried by the blood [12,13]. Yet despite his emphasis on physiological processes, Galen’s medical vision remained steeped in Stoic teleology, which asserted that every bodily organ had a divinely ordained purpose within a cosmic order. As a result, his innovations in physiological thinking did not translate into a surgical attitude. The heart, in Galen’s framework, retained its metaphysical sanctity—untouched, untouchable, and encased within an aura of moral and ontological reverence [12,13].

Indeed, Galenic doctrine perpetuated a dual vision of the heart: it was both measurable and mystical, corporeal and cosmological. This synthesis endured well into the anatomical treatises of the Renaissance, where even detailed visualizations of the heart rarely translated into operative intentions [12,13,14].

It should also be noted that the conception of the heart in ancient Greek thought, while symbolically significant, did not always confer absolute inviolability. Historical evidence indicates that dissection of the heart, both in animals and occasionally in human cadavers, was practiced in antiquity as part of anatomical inquiry. This suggests that while the heart was culturally esteemed, Greek physicians were not universally restrained by metaphysical prohibitions alone. Rather, the absence of operative cardiac interventions in this context reflects the decisive influence of technical limitations, including the lack of anesthesia, antiseptic methods, and effective hemostasis. Thus, unlike in ancient Egyptian or later Christian traditions where the heart carried an explicit sacred interdiction, in Greek medicine the barrier to cardiac surgery appears to have been shaped more by pragmatic constraints than by absolute cultural taboo [15,16,17,18].

**Figure 1:**
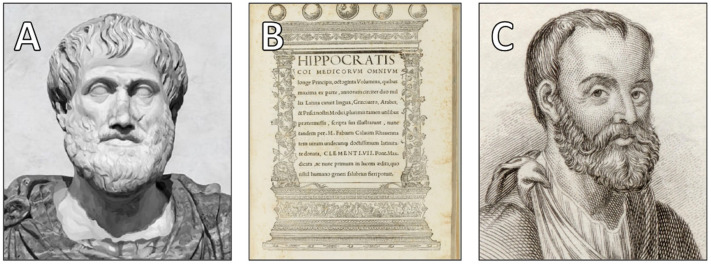
(**A**). Portrait of Aristotle (384–322 BC), a Greek philosopher whose ideas shaped early anatomical and metaphysical conceptions of the heart. (**B**). Octoginta Volumina, Latin edition of the Hippocratic Corpus translated by Marcus Fabius Calvus, 16th century (Courtesy of the National Library of Medicine, Bethesda, MD, USA). (**C**). Aelius Galenus (Galen of Pergamon), c. 129–216 AD, a Greek physician, surgeon, and philosopher and one of antiquity’s most influential physicians (Courtesy of Wellcome Collection, London, UK).

Thus, in classical antiquity, the heart was positioned not only as anatomically central but as ontologically inviolable—a locus of circulation, yes, but more profoundly, the sanctified core of human being. The foundational views of Aristotle, Hippocrates, and Galen (Figure 1) established a cardiocentric worldview that dominated classical and medieval medical thought, positioning the heart as both biological and moral epicenter.

These examples underline that views on the heart were neither monolithic nor static across time and culture. In the Greek and Roman eras, conceptions of the heart were closely tied to philosophy and physiology, and while symbolic weight limited the extent of intervention, it did not entirely prohibit dissection, as Galenic and Alexandrian practices demonstrate. By contrast, within later Jewish and Islamic traditions, the heart acquired a deeply sacred role that extended beyond symbolic significance into direct prohibition of invasive inquiry. This historical variability highlights that cultural and religious frameworks exerted diverse influences; in some contexts, they merely constrained investigation, while in others they categorically forbade it.

### 3.2. The Heart in Religious Thought

**Figure 2:**
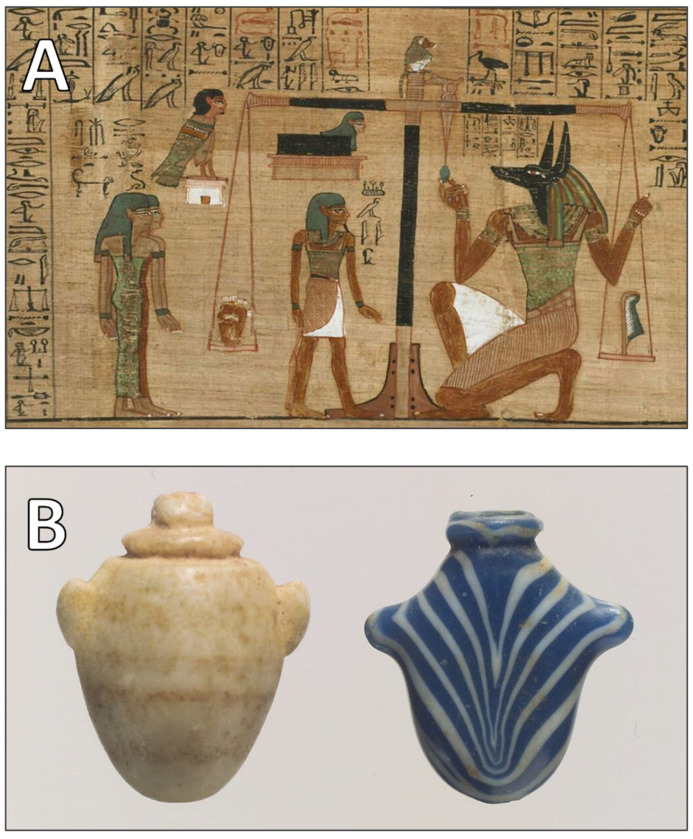
(**A**). Depiction of the Weighing of the Heart ritual from the Papyrus of Ani, c. 1275 BC. This scene symbolizes moral judgment in the afterlife, where the heart is weighed against the feather of truth. (Courtesy of The Trustees of the British Museum, London, UK). (**B**). Heart amulets from the New Kingdom, ca. 1550–1186 BC. These were placed on mummies to protect the heart and ensure a favorable judgment. (Courtesy of The Metropolitan Museum of Art, New York, NY, USA).

Perhaps the most metaphysically oriented interpretation of the heart appears in ancient Egyptian belief, as recorded in funerary texts such as the Book of the Dead. The ib—the metaphysical heart—was weighed against the feather of Ma’at to assess the moral integrity of the deceased. This judgment ritual, dating back as far as 1550 BCE, underscores the idea that the heart contained the ethical essence of the individual [3,19]. Any harm to the heart, even post-mortem, was thought to jeopardize the soul’s passage into eternity. Recent studies in medical anthropology confirm that the ib was not regarded metaphorically but as the literal seat of moral identity—inscribed with the record of a lifetime’s conduct [20,21]. This ancient belief system is vividly illustrated in funerary imagery and heart amulets from the New Kingdom period (Figure 2), which reflect the moral weight attributed to the heart and its essential role in one’s passage to the afterlife.

In Greek mythopoetic thought, as preserved in the Homeric epics, the chest—particularly through the concept of thymos—was imagined as the wellspring of spirit, anger, and intention. While thymos is not anatomically synonymous with the heart, its consistent localization in the thorax contributed to an early affective geography of the body [22,23,24]. Bruno Snell has argued that this mapping of emotional life onto bodily regions functioned as a proto-phenomenology of moral experience. This embodied view of emotion has found resonance in contemporary neurophilosophy, which revisits the somatic foundations of ancient affective models [25].

During the Byzantine era, the heart continued to be understood as more than a physiological organ; it was also a spiritual and emotional center. Drawing from Greco-Roman medical traditions and Christian theology, Byzantine physicians and scholars regarded the heart as the seat of the soul, emotions, and moral consciousness. It was believed to be intimately tied to divine presence and human virtue, playing a pivotal role in spiritual discernment and ethical decision-making. Although medical writing of the period increasingly reflected empirical observation, it retained this dual framework—viewing the heart simultaneously as a biological pump and a vessel of inner life and divine truth. This synthesis helped shape the holistic anthropological vision of the human being in Byzantine thought [26].

**Figure 3:**
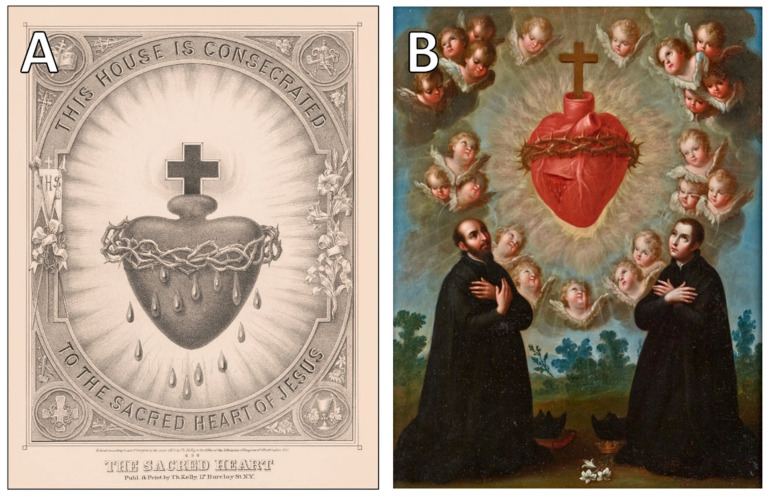
(**A**). The Sacred Heart, engraving by Thomas Kelly, 1874. An iconic representation of divine love and compassion in Catholic tradition (Courtesy of the Library of Congress, Washington, DC, USA). (**B**). The Adoration of the Sacred Heart with Saints Ignatius Loyola and Louis Gonzaga, c. 1770, by José de Páez. A devotional painting exemplifying the emotional and theological significance of the heart (Courtesy of the Denver Art Museum; Gift of the Collection of Frederick and Jan Mayer. Denver, CO, USA).

In the Christian tradition, more broadly, the symbolic weight of the heart was not diminished by scientific inquiry; rather, it was intensified. From early patristic theology to the mystical writings of the Middle Ages, the heart became a representation not only of virtue, but of divine immanence. Contemporary theological research interprets this shift as part of a broader movement from apophatic (non-representational) theology to affective devotional practices, increasingly centered on visceral imagery and emotional embodiment [27,28]. By the 17th century, this symbolic trajectory culminated in the formalization of the Sacred Heart cult, particularly through the revelations of Saint Margaret Mary Alacoque. The Sacred Heart came to signify not only Christ’s suffering but also His innermost being—rendered both anatomically visible and theologically radiant. In such a framework, any surgical intervention on the heart would not merely be a medical act, but a profoundly sacrilegious one [29]. The enduring iconography of the Sacred Heart, as seen in devotional engravings and paintings from the 18th and 19th centuries (Figure 3), reveals the heart’s elevation to a site of divine love and sacrificial virtue in Christian consciousness.

Comparable perspectives can be found across various world religions. In Islamic theology, the qalb is understood as the center of understanding, sincerity, and receptivity to divine guidance. The Qur’an frequently references the heart as the vessel through which God’s truth is recognized. As Seyyed Hossein Nasr has noted, the qalb is not merely symbolic, but ontologically real—a spiritual compass of the soul [30]. This view continues to inform contemporary Islamic bioethics, where the heart remains a privileged site of divine-human interface [31].

Similarly, in Jewish religious law, the integrity of the heart carried juridical significance. The Halakhic requirement to bury the body whole, including the heart, effectively prohibited dissection or autopsy for much of antiquity and the medieval period. Any tampering with the heart, especially post-mortem, was considered a desecration of bodily sanctity [32].

In all these religious traditions—Egyptian, Greek, Christian, Islamic, and Jewish—the heart is consistently portrayed as the locus of truth, judgment, vision, and transcendence. To cut it open, then, was never simply an act of scientific exploration, but a transgression of sacred boundaries—the sacrilegious gesture par excellence.

## 4. Early Surgical Practices: Approaching but Avoiding the Heart

Every part of the human body has historically been perceived through a particular lens of vulnerability—a perception shared even within the clinical gaze. Yet in ethical and cultural traditions, the heart emerged as the supreme locus of fragility—not due to its anatomical delicacy, but because it embodied an inviolable moral and symbolic gravity. The classical Hippocratic principle primum non nocere—“first, do no harm”—assumed particular weight in relation to the heart. This ethos has been shown to operate disproportionately in cardiac contexts, where the symbolic charge of the organ amplified the uncertainty of intervention [4,33,34]. The very notion of injuring the heart was as unthinkable as hurling the soul into Erebus—a conceptual repulsion that helps explain why cardiac intervention remained unapproachable for most physicians prior to the 19th century.

Religious prohibitions, steadfast and epistemically impermeable, left little room for the scientific imagination to expand its operative frontiers into the sanctified terrain of the human form. In Christian doctrine, particularly within the scholastic theology of the 13th century, the human body was regarded as a sacred vessel—not to be violated, even in the pursuit of healing or knowledge. Historians of medieval medicine have noted that such theological perspectives directly impeded the institutional development of anatomical dissection [4,35]. The heart, in this worldview, was not only sacred but emblematic—representing the suffering and love of Christ, and in some mystical accounts, the very locus of divine immanence. As Caroline Walker Bynum has shown, medieval Christian mysticism frequently associated the hearts of saints with miraculous incorruptibility, transforming the organ into the ultimate sign of divine favor and holiness [36].

Similarly, in Islamic jurisprudence, the qalb retained legal and spiritual sanctity even after death. Classical Islamic law prohibited post-mortem examinations except under exceptional justification, and even then, any intervention involving the heart was viewed as a serious violation of human dignity and divine order [31].

Contemporary research in Jewish bioethics likewise affirms the historical absolutism surrounding bodily integrity, particularly concerning the heart. Jewish halakhic tradition, rooted in Talmudic law and later codified by figures such as Maimonides, required the burial of the body in its entirety—thereby forbidding dissection or autopsy for centuries. This effectively ruled out post-mortem surgical experimentation well into the modern period [32,37]. The heart, in particular, could not be repurposed for secular aims, no matter how noble. Its proper place was deep within the chest, to be returned to the earth whole—awaiting eschatological fulfillment. It remained untouchable, as sacrosanct as the pupil of the eye.

## 5. Scientific Challenges Reinforcing Ethical Hesitations

Even in a hypothetical world devoid of religious and moral prohibitions, the heart would still have remained a forbidden zone—not because of its sacred status, but due to profound technical limitations that, until relatively recently, proved insurmountable. Anesthesia was unknown until the mid-19th century, and the absence of pain control made thoracic exploration nearly impossible, even in cases of acute necessity [38]. Any attempt at thoracic surgery was a torturous ordeal—one that often ended before completion, as patients succumbed to unbearable pain or traumatic shock [39].

Antiseptic protocols were likewise nonexistent. Before Joseph Lister’s introduction of antisepsis in the 1860s, even minor internal procedures carried the risk of catastrophic infection [40]. Recent historical analyses affirm that the lack of sterile technique rendered thoracic interventions practically suicidal during the pre-Listerian era [39]. The thoracic cavity, dense with critical vasculature and largely uncharted, was not merely anatomically complex—it was forbidding. It demanded extraordinary courage and precision long before it could even be cautiously explored.

Early anatomists such as Andreas Vesalius, despite their monumental advances in human dissection and anatomical accuracy, did not conceive of the heart as an operative site. In his seminal work De Humani Corporis Fabrica (1543), Vesalius depicted the heart with extraordinary detail—yet it remained an object of observation, not a target of intervention [41]. His anatomical illustrations, although groundbreaking, perpetuated a contemplative gaze: the heart was to be admired and understood, not touched or altered [42]. For observation to give way to intervention, several conceptual and technological thresholds still had to be crossed.

**Figure 4:**
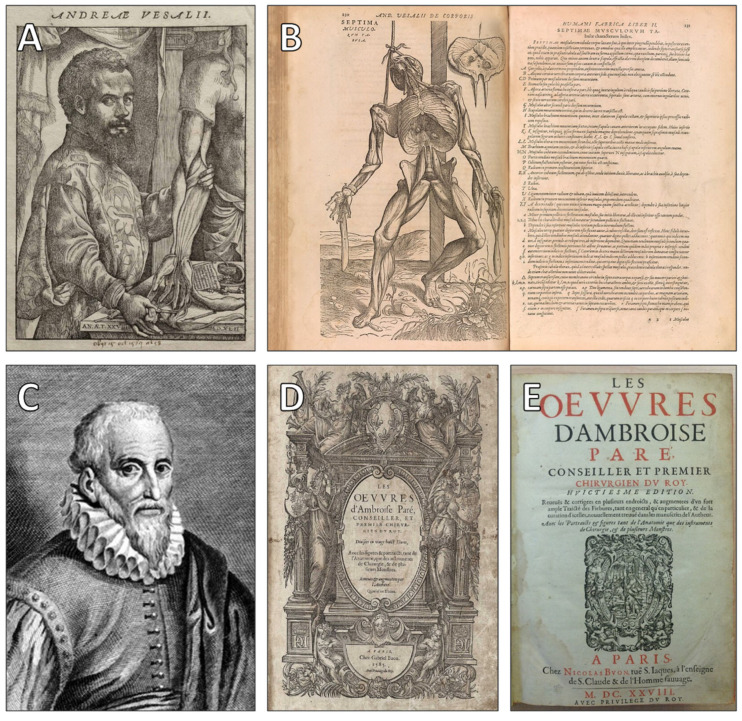
(**A**) Andreas Vesalius (1514–1564), an anatomist whose work revolutionized the understanding of human anatomy (Courtesy of the U.S. National Library of Medicine, Bethesda, MD, USA). (**B**) De Humani Corporis Fabrica (1555), an anatomical atlas by Vesalius, illustrated by John of Calcar (Courtesy of The Metropolitan Museum of Art, New York, NY, USA. Gift of Dr. Alfred E. Cohn, in honor of William M. Ivins Jr., 1953). (**C**) Ambroise Paré (c. 1510–1590), a pioneer of battlefield medicine and surgery. (Public domain; courtesy of Bibliothèque nationale de France, Paris, France). (**D**,**E**) Title page and frontispiece of Oeuvres d’Ambroise Paré (1628), showcasing his contributions to surgical technique (Courtesy of the Wellcome Collection, London, UK).

The earliest meaningful steps in this direction came not from deliberate experimentation, but from battlefield exigencies. In the 16th century, Ambroise Paré and other military surgeons occasionally encountered thoracic injuries, yet even in these moments, the aim was stabilization, not direct cardiac access [43,44]. When soldiers suffered wounds near the heart, they were often left to die—not out of clinical indifference, but because the heart was considered physiologically unreachable. It was not sacred in this context; it was simply beyond operative reach. Contemporary surgical records consistently described the heart as “fortified by nature”—a phrase that reveals the conceptual frame in which the heart remained sealed off from human intervention [45]. Figures such as Vesalius and Paré (Figure 4) advanced anatomical knowledge and surgical technique, yet still approached the heart as an object of contemplation rather than intervention—a sign of the enduring anatomical and ethical threshold it represented.

In this way, technical limitations did not merely accompany ethical prohibitions—they intensified them. The heart was not only metaphysically sacred; it was logistically untouchable.

## 6. Shifts in Medical Thinking: Toward the Possibility of Heart Surgery

As anatomical knowledge deepened and surgical ambition began to chafe against longstanding taboos, a conceptual shift became increasingly inevitable. The convergence of scientific discovery and philosophical realignment would soon challenge both the metaphysical and logistical barriers that had long rendered the heart untouchable.

The first decisive rupture occurred in the early 17th century with William Harvey’s Exercitatio Anatomica de Motu Cordis et Sanguinis in Animalibus (1628), in which he described the circulation of blood. With this discovery, the heart was recontextualized: no longer the mystical furnace of pneuma, it became a pump—precise, mechanical, and measurable [13,46]. This mechanistic reinterpretation laid the intellectual foundation for a physiology increasingly decoupled from metaphysical residues [47]. While Harvey’s reframing did not yet render the heart operable, it redirected the epistemic gaze: the heart could now be envisioned as a system of functions to be modeled, quantified, and eventually acted upon.

In Harvey’s wake, scientific culture in Europe entered a crescendo, and metaphysical fixations—once unassailable—began to teeter toward obsolescence. The Enlightenment ushered in a new body paradigm: no longer a vessel of divine mystery, the human body was reimagined as a repairable machine. This secularization of anatomy and pathology is well documented in the medical archives of the era [48]. The Enlightenment body, now intelligible and dissectible, stood increasingly open to intervention.

Anatomists such as Giovanni Battista Morgagni in the 18th century helped further this shift by correlating pathological findings with specific clinical symptoms, thereby anchoring the medical gaze more firmly in the material substrate of disease [49]. Yet even this progress, significant as it was, did not itself generate the radical mental shift cardiac surgery would require—one forged in equal parts boldness, caution, and reverence [50].

By the 19th century, technological improvements brought the heart incrementally closer to clinical accessibility. Laënnec’s invention of the stethoscope in 1816 allowed physicians to “hear” the heart without touching it—a new diagnostic intimacy that nonetheless fell short of overcoming the psychological and technical distance still separating medicine from cardiac intervention [51].

**Figure 5:**
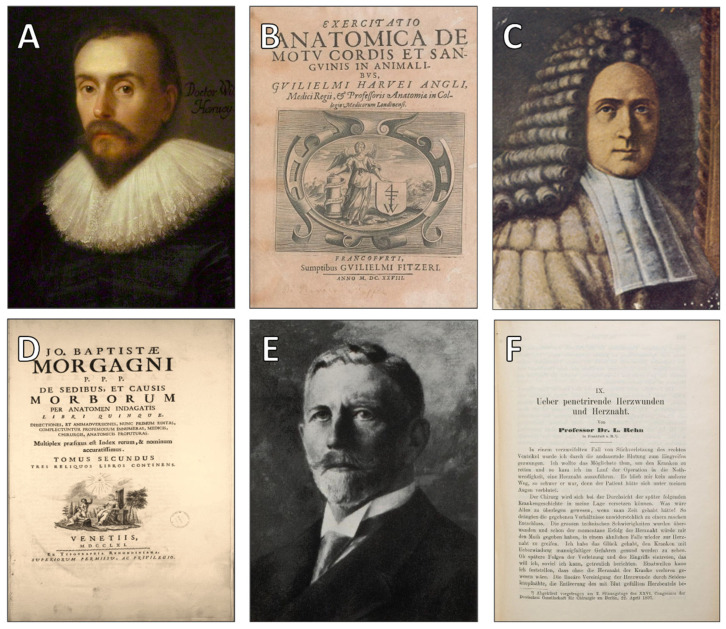
(**A**). William Harvey (1578–1657), an English physician who first described blood circulation. (Public domain; courtesy of the National Portrait Gallery, London, UK). (**B**). Title page of Exercitatio Anatomica de Motu Cordis et Sanguinis in Animalibus (1628), Harvey’s groundbreaking treatise (Courtesy of the Wellcome Collection, London, UK). (**C**). Giovanni Battista Morgagni (1682–1771), father of anatomical pathology. (**D**). De Sedibus (1765), Morgagni’s major work correlating pathology with clinical symptoms (Courtesy of the National Library of Medicine, Bethesda, MD, USA). (**E**). Ludwig Rehn (1849–1930), a German surgeon who performed the first successful cardiac repair (Public domain; courtesy of Deutsches Medizinhistorisches Museum, Ingolstadt, Germany). (**F**). Original report of Rehn’s cardiorrhaphy for a penetrating heart wound (Courtesy of the German Medical Archives, Koblenz, Germany).

The long-standing prohibition was not decisively breached until 1896, when Ludwig Rehn, a German surgeon, performed what is widely regarded as the first successful cardiac surgery by suturing a stab wound to the right ventricle [52,53]. Rehn’s intervention marked the inaugural moment of modern cardiac surgery, even if its replication remained rare for many years [54]. His scalpel tore through more than skin and muscle—it severed a prohibition that had withstood millennia. With the work of Harvey, Morgagni, and Rehn (Figure 5), the epistemic and technical foundations of modern cardiology were laid—marking the transition from reverence to repair, and from symbolic resistance to surgical intervention.

Yet this act was not universally celebrated. While it ignited optimism within medical circles, it also provoked moral unease. The surgeon’s hand, once barred from the sanctum of the heart, now raised questions not only of possibility but of propriety. As Thomas Schlich observes, early cardiac surgeons were cast either as heroic pioneers or blasphemers in scrubs—depending on whether one regarded the heart as mere tissue or as temple [55]. This symbolic ambivalence persists even in today’s public perception of invasive cardiac procedures.

The paradigm had shifted irreversibly. The ancient axioms—once shielded by theology, fear, and tradition—could no longer withstand the momentum of scientific advancement. As surgical technique evolved, so too did the ontological status of the heart. Medicine had begun its full transition: from mystery to mechanism.

## 7. Modern Era: Redefining Ethics in Cardiothoracic Surgery

The 20th century brought a cascade of innovations that redefined the conceptual and ethical landscape of cardiology and surgery. Ironically, many of these breakthroughs were accelerated by the urgencies of war, as technological advancement collided with existential need. In this crucible of crisis and innovation, bioethics emerged not as an abstract discipline, but as an institutional imperative. The convergence of wartime medical progress and the postwar codification of ethical standards marked the birth of modern surgical bioethics [56].

As cardiothoracic surgery entered the medical mainstream, a distinct moral framework began to crystallize around it. The cardiac surgeon was no longer viewed as a moral transgressor or reckless experimentalist; the stigma surrounding surgical boldness began to recede. Instead, the surgeon came to be seen as a figure of sacrificial precision, operating at the threshold of life and death in defense of the only sanctity medicine could still credibly invoke: the preservation of life itself [57].

A central pillar of this ethical shift was the emergence of informed consent as a foundational principle in medical practice. In the aftermath of the Nuremberg Code (1947) and subsequent declarations such as the Helsinki Declaration (1964), the patient was no longer regarded as a passive subject or a vessel of paternalistic judgment. Instead, the patient was recognized as a moral agent—autonomous, sovereign, and entitled to self-determination [58,59,60]. Particularly in high-risk fields like cardiac surgery, informed consent was elevated beyond legal necessity to moral safeguard [61]. The decision to operate on the heart became not just a clinical matter, but an existential dialog between physician and patient.

Meanwhile, cardiac interventions began to resonate with symbolic and cultural overtones. In popular media and public imagination, the “open heart” was increasingly reframed as a site not only of surgical achievement but of emotional transformation and existential vulnerability [62]. The heart—once sacrosanct and off-limits—became a metaphor for courage, authenticity, and even redemption. Medical imagery blurred with poetic symbolism: the open chest was no longer only an anatomical event, but a semiotic one. In this reframing, the leap into cardiac surgery rivaled humanity’s first glimpse of Earth from the Moon—a moment in which human limits were not just tested, but transcended [63,64].

Yet despite these advances, the ethical terrain remained fraught. Medical progress brought with it a new layer of controversy. As biopolitical discourse gained momentum, questions emerged about the allocation of resources, post-operative quality of life, and the ethics of prolonging life under compromised conditions. What constituted a “life worth saving” could no longer be assessed solely through physiological indicators. Instead, it became a contested metric—one that weighed prognostic data, cost-effectiveness, and the psychological burden of survival [65,66]. The once-unquestioned virtue of saving a life became a moral calculus in its own right.

Among the most consequential innovations in the history of cardiac surgery was the development of the heart–lung machine by Dr. John Gibbon in 1953. By enabling extracorporeal circulation, this device made open-heart surgery technically feasible for the first time. Yet this leap in capability also raised profound ethical concerns. Was it morally permissible to suspend the natural function of vital organs and sustain life artificially? Critics feared a creeping dehumanization—a potential overreach of medicine into domains once reserved for fate or divinity. The debates surrounding artificial life support reflected broader cultural anxieties about technological power and the shifting boundaries of what it means to intervene in life and death [63,64,67].

These dilemmas were magnified further by the advent of heart transplantation. When Christiaan Barnard performed the first successful human heart transplant in 1967, he reignited metaphysical debates in clinical form. Where, exactly, is the boundary between life and death? Whose identity inhabits a transplanted heart? The movement of a heart from one body to another raised ontological questions about selfhood, continuity, and embodiment [68]. Transplant ethics, still evolving today, continues to grapple with these questions—particularly as some transplant recipients report psychological transformations that seem to echo the identity of the donor [69,70].

The symbolic and existential questions raised by heart transplantation have also been explored in the medical humanities. Richard Selzer’s celebrated short story Whither Thou Goest reflects on the persistence of personal identity through the transplanted heart, posing metaphysical and moral questions about the relationship between organ, donor, and recipient. Heart transplantation challenges the boundaries between physiology, selfhood, and morality, demanding a reconsideration of how medical practice intersects with human meaning [71]. These reflections demonstrate that even after transplantation became technically feasible, the heart continued to function as a powerful symbol within cultural and ethical discourse.

The total artificial heart (TAH) represents a unique case in which the patient survives without a biological heart. This development has challenged traditional definitions of life and death [72]. The widespread use of continuous-flow devices, such as modern left ventricular assist devices (LVADs), has introduced a state in which patients can survive without a palpable pulse [73]. Since the pulse was historically regarded as a necessary sign of life, these technologies compel a re-evaluation of symbolic, physiological, and ethical conceptions of vitality. In this respect, mechanical replacement devices extend the historical trajectory of cardiac surgery beyond the resolution of technical barriers, confronting deep-rooted cultural assumptions about the heart as the essential locus of life. Hansen & Stevens describe how LVAD-related pulselessness contradicts cultural and literary traditions that equate pulse with life [74]. Miles et al. [75] and DeMartino et al. [72] discuss ethical dilemmas surrounding TAH use and its withdrawal, highlighting how life definitions are challenged when the native heart is completely replaced. Finally, physiological work such as Baric et al. explores how non-pulsatile flow functions in the body, supporting survival despite loss of palpable pulsation [76].

The sacred, it seems, did not disappear. It merely shifted—from theology to bioethical deliberation. The scalpel, once barred by religious interdiction, was now tasked with navigating a dense terrain of symbolic, emotional, and existential meanings. In this new ethical landscape, salvation and violation stood side by side, each shaped by radically different premises—but both intrinsic to the modern surgical imagination [66].

## 8. Discussion: Between Tissue and Meaning, Between Surgery and Soul

The historical trajectory of cardiac surgery—from a once-unthinkable transgression to a core domain of medical expertise—reveals how the boundary between biology and spirit is not merely theoretical but lived. What was once considered a desecration is now a routine intervention executed with the utmost precision. Yet the heart has not shed its symbolic charge. Ethical ambiguities resurface whenever medical progress attempts to reframe humanity through mechanistic or deconstructivist models [63,64,77].

It would be intellectually naïve to assume that the heart has been entirely stripped of its metaphysical residues. Despite its complete clinical integration, it remains saturated with cultural and emotional significance. Colloquial expressions—“broken heart,” “heartfelt,” “he has no heart”—are not just linguistic fossils, but ongoing psychic investments in the organ’s symbolic centrality [7,78].

The depth of this symbolic persistence becomes especially apparent in questions that flirt with literalism: could part of a person’s identity remain anchored in the heart after death? From a scientific perspective, such a belief is untenable. Contemporary neuroscience has definitively located consciousness in the brain. Yet the notion that subjectivity is partially embodied—distributed across affective and visceral systems—has gained traction in phenomenology [79], affective neuroscience [80], and cardiac psychology [81]. Research increasingly confirms that interoceptive networks involving the heart play active roles in emotional salience and embodied decision-making [77,82].

The heart, with its intrinsic nervous system and role in neurocardiac feedback loops, may not be the seat of reason—but it is no mere pump. The phenomenon of so-called cardiac memory, observed in transplant recipients who report shifts in emotion or personality, has fueled a small but persistent body of literature [83]. While many of these reports are dismissed as psychological projection, their consistency challenges the reductionist narrative of pure biological function. These accounts may not offer metaphysical proof, but they unsettle the mechanistic orthodoxy that often underpins scientific discourse [84,85].

Cardiac surgery may have achieved the heart’s secularization in terms of operability, but its symbolic aura continues to mark the limits of biotechnological sovereignty. The heart still resides at a liminal frontier—between physiology and mystery, between life and its cessation. Those who operate on it become, in effect, ritual actors in a clinical drama where tissue and transcendence converge.

Yet this discussion must resist the seductions of mysticism. The neurocentric model of cognition remains the most robust explanatory framework available. If there is a locus of selfhood, it resides in the cortex, where reflection, judgment, and memory take shape. Still, the body—and the heart, in particular—cannot be dismissed as a passive substrate. It participates in the enactive matrix through which subjectivity is experienced and lived.

In this sense, the heart retains its singular value as a source of affective meaning. This significance is far from trivial. It demands humility from those who intervene upon it, reminding us that no incision—however sterile—is devoid of existential consequence. This perspective aligns with the principles of narrative medicine, which emphasize the semantic and symbolic implications of clinical acts [86]. To touch the heart, even in the era of precision medicine, is to touch a narrative—not merely a muscle.

Even if myth no longer governs the conceptual edges of medical thought, the heart remains poised at the intersection of biology and story, of mechanism and metaphor. It is here, on this liminal threshold, that physiology and semantics continue their entangled dance. Biosemiotic theory has begun to explore such spaces, framing the heart as a boundary organ of meaning-generation—where sense, symbol, and substance intertwine [87]. The scalpel, then, though cold in function, must never relinquish its contemplative vocation. It is this dual demand—precision and reverence—that ultimately defines cardiac surgery as both a technical act and an existential gesture [88].

Although the present review focuses on Western cultural, religious, and scientific traditions, it is important to acknowledge that rich and sophisticated conceptions of the heart also developed in Eastern medical and philosophical systems. In Chinese philosophy and medicine, the heart (xin) was not only regarded as a central physiological organ but also as the locus of thought, feeling, and morality, unifying cognition and emotion into a single concept of heart–mind [89]. Similarly, in Indian Ayurvedic medicine, the heart (hridaya) was described as the seat of life, consciousness, and emotion, as well as the central regulator of circulation, with classical texts outlining both symbolic and functional dimensions of cardiac activity [90,91,92]. These perspectives highlight the cross-cultural universality of attributing symbolic and existential weight to the heart. A detailed analysis of Eastern traditions lies beyond the scope of this article, which remains centered on Western developments, but their acknowledgement underscores the global significance of the heart as a nexus of physiology, identity, and meaning.

Looking ahead, future perspectives on the ethical and symbolic dimensions of cardiac surgery must account for the accelerating integration of artificial intelligence, machine learning, and organ bioengineering into clinical practice. As technologies such as bioprinted hearts, neural-heart interface systems, and predictive surgical algorithms become more prevalent, the ontological status of the heart may once again shift—from organic to synthetic, from embodied to programmable. These developments will likely intensify ethical questions surrounding identity, autonomy, and the limits of human intervention. Moreover, interdisciplinary dialog between medicine, philosophy, theology, and cultural studies will be essential in navigating the evolving meanings of the heart in both clinical and existential registers. Far from being resolved, the tension between mechanism and meaning, intervention and reverence, is poised to deepen—inviting renewed reflection on what it means to heal, to touch, and ultimately, to live.

## 9. Limitations

This manuscript adopts a multidisciplinary and interpretive approach that necessarily carries certain limitations. First, the analysis is primarily historical and philosophical in nature, drawing from medical history, religious studies, anthropology, and cultural theory rather than from empirical clinical data. While this broad lens enriches the ethical and symbolic narrative surrounding cardiac surgery, it may limit the immediate applicability of conclusions to contemporary medical practice or policy.

Second, due to the scope and depth required to trace symbolic and ethical meanings across millennia, the discussion is necessarily selective. Certain religious traditions, cultural frameworks, or regional histories may have been underrepresented or omitted altogether. The focus was placed on major Western and Abrahamic traditions due to their historical influence on modern biomedical ethics, but further work could fruitfully expand this inquiry to include Eastern philosophies, Indigenous belief systems, and global south perspectives. It is important to note that while cultural and religious conceptions of the heart profoundly influenced medical practice in many societies, these perspectives were not universal. Certain traditions and communities may have placed less symbolic weight on the heart or approached it with more pragmatic medical attitudes. The analysis presented here therefore emphasizes dominant historical trajectories within Western and Abrahamic traditions, without claiming uniformity across all societies and eras.

Third, while reference has been made to psychological and phenomenological studies of post-transplant identity and cardiac memory, these areas remain scientifically controversial and methodologically limited. Interpretations of such reports were approached with caution, acknowledging the anecdotal nature of the evidence while highlighting their philosophical implications rather than their empirical validation.

Finally, the narrative nature of the manuscript does not follow a systematic review methodology. The sources were selected for their conceptual relevance and historical richness rather than through a standardized search protocol, which may result in selection bias. Future work could supplement this narrative exploration with quantitative studies, patient interviews, or clinician perspectives to deepen the empirical foundation of the ethical questions posed.

## 10. Conclusions

The evolution of cardiac surgery—from sacred taboo to clinical routine—illustrates the deep interplay between medicine, culture, and meaning. What once seemed a moral and metaphysical violation is now a symbol of medical achievement, yet the heart retains its symbolic weight. This manuscript has shown that the historical resistance to cardiac intervention stemmed not only from technical limits but from enduring cultural and ethical conceptions of the heart as the core of identity and spirit. As medical techniques advanced, so too did ethical frameworks, shifting the sacred into the realm of informed consent, autonomy, and bioethics. Still, contemporary debates—from heart transplantation to artificial life support—reveal that the heart remains more than a pump. It continues to function as a site where biology and narrative, physiology and symbolism, converge. In this sense, cardiac surgery occupies a unique space: both precise and profound, it reminds us that healing is never purely mechanical. To touch the heart is always, in some measure, to engage with what it means to be human.

## Data Availability

The data presented in this study are available upon request from the corresponding author.
